# Supplementary material for: Current Consumption of Traditional Cowpea-Based Dishes in South Benin Contributes to at Least 30% of the Recommended Intake of Dietary Fibre, Folate, and Magnesium
Source: Nutrients. 2023 Mar 7;15(6):1314. doi: 10.3390/nu15061314 (PMC10056862; doi:10.3390/nu15061314)
Supplement: Supplementary file 1 [file nutrients-15-01314-s001.zip › nutrients-2200710-supplementary.pdf]

Supplementary Materials

Table S1. Description of the nine traditional cowpea-based dishes.

	Cowpea-based dishes	Principal ingredients §	Description
**Stews**	Abobo	Cowpea	Boiled cowpea seeds that are almost pureed after cooking and still contain free water.
	Vêyi	Cowpea	Boiled cowpea seeds with no free water left after cooking
	Adowè	Dehulled cowpea	Dehulled cowpea seeds cooked in water.
**Doughnuts**	Ata	Dehulled cowpea	Fried cowpea dough made with dehulled seeds
	Ata-doco	Cowpea	Fried cowpea dough made with whole seeds
	Atační	Cowpea	Whole cowpea dough fried twice and stored in oil after frying
**Mixed dishes**	Atassi	Cowpea: Rice (1:5 to 2:7) Cowpea/Rice (w/ w)	Rice and cowpea cooked in water
	Abla	Maize: Cowpea: Palm Nut Extract or Red Palm Oil (3:3:4)	Paste made from steamed dehulled cowpea with red palm oil/palm nut extract and some cowpea seeds
	Djongoli	Cowpea:Maize:Oil (3:3:1)	Cowpea seeds and maize flour; sometimes cooked with red oil or palm nuts extract.

§: Information collected from (Madode et al. 2011).

Table S2. Reference mean values used to calculate the contribution of cowpea-based dishes to the recommended nutrient intakes (RNIs).

		Women	Men	All ǂ
Energy	Energy (kcal)	2200	2800	2500
Macronutrients	Protein (g)	54	54	54
	Fibre (g)	30	30	30
Minerals	Ca (mg)	1000	1000	1000
	Mg (mg)	220	260	240
	K (mg)	3510	3510	3510
	Zn [1] (mg)	9.8	14	11.9
	Fe [2] (mg)	58.8	27.4	43.1
Vitamins	Folate (µg)	400	400	400
	Thiamine (mg)	1.1	1.2	1.15

ǂ Mean values of RNIs for women and men. These values were used to calculate the contribution to RNIs. [1] RNIs for zinc, based on a bioavailability of 15%. [2] RNIs for iron, based on a bioavailability of 5%. References values were from: FAO-WHO, (2004) for energy; FAO and WHO, (2007) for proteins; ANSES, (2016) for fibre; FAO and WHO, (2004) for vitamins and minerals.

Table S3. Food atlas and FFQ validation using Spearman's correlations and the Bland-Altman method.

**Cowpea-based dishes**		**Food Atlas validation Weight record vs 24-h recall**					**FFQ validation FFQ vs Mean values of the three 24-h recalls**				
		**n**	**Spearman's correlation**		**Bland and Altman test (ratio %)**		**n**	**Spearman's correlation**		**Bland and Altman test (ratio %)**	
			R	p-value	Mean	[LLOA;ULOA]		R	p-value	Mean	[LLOA;ULOA]
Stews	Abobo	51	0.5	<0.001	108	[45;258]	99	0.42	<0.001	96	[32;282]
	Vêyi	31	0.64	<0.001	162	[65;400]	23	0.74	0.05	95	[39;233]
	Adowè	51	0.21	>0.05	75	[29;195]	18	0.62	>0.05	66	[23;191]
Dough nuts	Ata	51	0.85	<0.001	84	[52;138]	35	0.85	<0.001	95	[59;152]
	Ata-doco	34	0.71	<0.001	74	[41;134]	25	0.74	<0.05	82	[40;165]
	Ataclè	34	0.83	<0.001	128	[92;178]	7	na		na	
Mixed dishes	Atassi	52	0.38	<0.05	99	[47;208]	112	0.37	<0.001	94	[39;228]
	Abla	19	0.64	<0.05	77	[45;131]	5	na		na	
	Djongoli	51	0.41	<0.05	94	[39;228]	37	0.64	<0.05	86	[32;234]

[LLOA; ULOA]: Lower and upper limits of agreement at 95% confidence interval. na: Sample size was too small to perform the analysis.

Table S4. Influence of socio-economic and demographic factors on the daily quantities of cowpea-based dishes consumed during the week before the FFQ survey.

Area	Factors		Cowpea-based dishes (g, FW)									Cowpea seeds ǂ (g, FW)
			Stews			Doughnuts			Mixed dishes			
			Abobo	Vêyi	Adowè	Ata	Ata-doco	Ataclè	Atassi	Abla	Djongoli	
Rural	**Sample size**		496	77	35	122	188	35	440	85	213	
	**Commune**	**Adjohoun (reference)**										
		Allada	**-28.1****	4.4	6.1	**12.8***	2.8	6.0	-6.5	5.0	-2.8	**-10.3***
	**Education**	**None (reference)**										
		Primary school	-9.1	3.4	4.0	4.9	1.0	15.9	-37.2	4.0	6.1	-8.7
		Secondary school	-5.9	-7.1	19.7	12.2	-3.2	24.2	-8.8	-14.7	32.7	-3.7
		University	24.6		-	21.4	-10.3	-7.4	-27.1	-3.3	**102.5***	-1.7
	**Age**	**<27 years (reference)**										
		27–34 years	11.6	33.0	-13.6	-11.9	-2.3		**-35.5***	-31.2	7.8	-1.2
		35–44 years	9.4	18.3	-4.3	-7.1	-2.0	-0.4	**-40.6****	-0.4	8.0	5.4
		45–65 years	2.9	15.0	9.6	-2.8	-9.0	-23.6	**-70.8*****	-4.6	31.9	-2.7
	**Sex**	**Men (reference)**										
		Women	-13.0	-22.3	**23.1***	-0.2	-8.3	0.5	**-22.0***	**-17.6***	-0.06	**-9.2***
	**SEI 2**	**Low (reference)**										
		Middle	0.2	-2.2	0.02	-9.7	-6.6	7.3	12.2	0.4	-6.3	-3.4
		High	**-20.7***	-9.7	-4.8	-13.2	-5.5	5.0	-1.1	-4.2	**-51.3*****	**-17.1****
Urban	**Sample size**		441	142	150	350	92	38	444	30	95	
	**Education**	**None (reference)**										
		Primary school	2.0	18.8	17.6	**24.2****	**-62.1****	22.0	-3.2	**185.7****	14.2	**20.8***
		Secondary school	-2.1	-11.5	1.2	5.8	14.1	5.7	2.6	21.0	-1.2	-0.005
		University	5.4	-3.5	-10.0	11.0	-12.1	-31.6	0.9	-13.2	-13.9	-12.5
	**Age**	**<27 years (reference)**										
		27–34 years	10.0	5.3	2.5	1.4	33.5	-32.2	-10.3	-15.5	2.0	6.7
		35–44 years	7.8	10.4	3.2	-14.8	31.2	**-53.2****	-14.8	5.4	7.9	-0.25
		45–65 years	3.7	1.1	0.4	-12.4	21.1	**-54.1***	-17.0	-49.9	8.8	-7.8
	**Sex**	**Men (reference)**										
		Women	**-49.1*****	**22.2***	-4.4	**-12.0***	-11.2	**35.9***	**-18.0***	25.9	-4.3	**-35.0*****
	**SEI 1**	**Low (reference)**										
		Middle	-1.0	3.8	9.6	11.7	**41.1***	-28.3	-21.2	56.1	-12.8	9.0
		High	-9.1	2.2	**15.6***	**17.3***	20.6	8.6	**-27.4***	-0.2	**-30.5***	21.9

ǂ Quantities of dishes were converted into quantities of cowpea seed equivalent. SEI 1: Socio-economic index in the urban area. SEI 2: Socio-economic index in the rural areas. Values are GLM coefficients and p-values are shown when significant (*: 0.01 < p ≤ 0.05; **: 0.001 < p ≤ 0.01; ***: p ≤ 0.001).

Table S5. Comparison of the nutritional composition of the nine cowpea-based dishes sampled at different street food vendors (100 g of edible portion).

Group	Dishes	Producers	Protein (g)	Lipid (g)	Available carbohydrate (g)	Ash (g)	Energy (Kcal)	Ca (mg)	Fe (mg)	Mg (mg)	Zn (mg)	K (mg)	Folate (µg)	Thiamine (µg)
**Stews**	**Abobo**	P1	7.1 ±0.1	0.5 ±0.0	17.2 ±0.0	1.4 ±0.0	113 ± 0	34.5 ±2.2	1.6 ±0.0	48.6 ±1.7	1.1 ±0.1	333.5 ±14.8	53.7 ±0.2	43.1 ±1
		P2	6.0 ±0.1	0.5 ±0.0	14.3 ±0.0	1.3 ±0.0	97 ± 0	23.2 ±2.4	1.0 ±0.1	32.7 ±4.2	0.8 ±0.1	245.7 ±31.6	61.1 ±4.4	58.8 ±2
		P3	5.7 ±0.1	0.6 ±0.0	11.3 ±0.1	2.0 ±0.0	85 ± 0	33.2 ±1.8	1.6 ±0.0	36.9 ±2.8	0.7 ±0.0	236.6 ±22.3	53.0 ±2.1	28.7 ±0
		P-value	*<0.001*	*Ns*	*<0.001*	*<0.001*		*0.02*	*0.004*	*0.03*	*0.04*	*0.05*	*0.02*	*<0.001*
	**Vêyi**	P1	7.0 ±0.0	0.6 ±0.0	14.4 ±1.0	1.6 ±0.0	105 ± 0	21.7 ±0.7	1.6 ±0.0	47.0 ±1 .8	1.0 ±0.1	328.9 ±19.3	73.9 ±3.7	114.6 ±4
		P2	7.9 ±0.0	0.4 ±0.0	18.8 ±0.0	2.2 ±8.7	125 ± 0	31.6 ±1.6	1.4 ±0.0	42.3 ±1.3	1.1 ±0.0	277.4 ±6.0	88.8 ±4.5	117.9 ±8
		P3	7.5 ±0.0	0.7 ±0.0	16.9 ±0.0	1.8 ±0.0	119 ± 0	30.3 ±0.5	1.4 ±0.0	49.7 ±1.4	1.2 ±0.0	361.8 ±9.9	78.8 ±4.0	36.2 ±2
		P-value	*<0.001*	*<0.001*	*<0.001*	*<0.001*		*0.004*	*0.01*	*0.04*	*0.02*	*0.02*	*0.01*	*0.001*
	**Adowè**	P1	7.4 ± 0.0	0.2 ± 0.0	16.2 ± 0.1	1.5 ± 0.0	106 ± 0	25.5 ± 1.2	1.3 ± 0.1	39.5 ± 1.3	1.0 ± 0.0	253.7 ± 11.6	57.2 ± 2.2	47.9 ± 0
		P2	4.8 ± 0.2	0.5 ± 0.0	10.7 ± 0.2	1.5 ± 0.0	76 ± 0	17.5 ± 1.1	0.9 ± 0.1	31.6 ± 1.9	0.7 ± 0.0	220.4 ± 15.0	45.8 ± 3.5	2.2 ± 0
		P3	6.7 ± 0.1	0.6 ± 0.0	14.1 ± 0.0	1.3 ± 0.0	99 ± 0	34.1 ± 1.6	1.0 ± 0.1	39.3 ± 1.9	1.0 ± 0.0	299.2 ± 14.2	92.4 ± 12	35.1 ± 0
		P-value	*<0.001*	*<0.001*	*<0.001*	*0.01*		*0.002*	*0.04*	*0.03*	*0.01*	*0.02*	*<0.001*	*<0.001*
**Dough nuts**	**Ata**	P1	7.0 ± 0.0	16.3 ± 0.3	18.1 ± 0.3	2.2 ± 0.0	257 ± 1	21.7 ± 0.8	4.6 ± 0.0	49.6 ± 1.4	1.1 ± 0.0	328.3 ± 9.8	80.9 ± 23	117.4 ± 28
		P2	8.0 ± 0.0	13.3 ± 0.3	20.7 ± 0.3	2.1 ± 0.1	244 ± 2	25.0 ± 1.5	4.0 ± 0.1	49.8 ± 2.7	1.2 ± 0.1	352.6 ± 20.9	79.0 ± 7.2	146.9 ± 3
		P3	7.4 ± 0.1	29.0 ± 1.2	17.2 ± 1.1	1.8 ± 0.0	368 ± 6	37.8 ± 0.2	4.0 ± 0.5	49.8 ± 0.2	1.2 ± 0.0	318.0 ± 3.8	50.8 ± 7.0	135.5 ± 2
		P-value	*<0.001*	*<0.001*	*0.03*	*0.006*		*0.001*	*Ns*	*Ns*	*Ns*	*Ns*	*<0.001*	*Ns*
	**Ata doco**	P1	7.6 ± 0.1	18.6 ± 0.4	21.1 ± 0.2	1.7 ± 0.1	294 ± 2	28.0 ± 1.1	3.7 ± 0.2	56.2 ± 2.0	1.4 ± 0.1	369.7 ± 16.1	52.4 ± 2.7	132.3 ± 10
		P2	9.1 ± 0.1	23.3 ± 0.2	21.5 ± 0.1	2.6 ± 0.0	345 ± 1	29.4 ±0.8	3.0 ± 0.2	62.0 ± 2.4	1.6 ± 0.0	434.1 ± 16.1	86.3 ± 9.6	165.5 ± 3
		P3	9.7 ± 0.1	7.6 ± 0.8	23.2 ± 1.0	2.4 ± 0.1	213 ± 4	38.8 ±5.1	5.5 ± 1.0	63.7 ± 8.0	1.4 ± 0.2	472.1 ± 64.7	53.4 ± 5.3	109.5 ± 9
		P-value	*<0.001*	*<0.001*	*Ns*	*0.001*		*Ns*	*0.05*	*Ns*	*Ns*	*Ns*	*0.001*	*0.01*
	**Ataclè**	P1	10.1 ± 0.0	34.5 ± 0.1	24.7 ± 0.1	2.0 ± 0.0	464 ± 0	24.4 ± 0.2	3.2 ± 0.1	67.9 ± 0.4	1.7 ± 0.0	509.2 ± 1.1	55.8 ± 3.0	148.5 ± 15
		P2	10.0 ± 0.0	28.6 ± 0.4	25.1 ± 0.2	2.3 ± 0.1	411 ± 2	37.8 ± 1.6	15.9 ± 6.2	73.9 ± 2.7	1.7 ± 0.1	516.3 ± 21.6	100 ± 8.6	197.5 ± 3
		P3	9.6 ± 0.2	36.8 ± 0.5	21.8 ± 0.3	2.4 ± 0.0	471 ± 3	38.1 ± 3.8	4.9 ± 0.4	63.4 ± 6.2	1.4 ± 0.2	473.4 ± 47.8	96.8 ± 5.2	178.8 ± 2
		P-value	*0.04*	*<0.001*	*0.001*	*0.02*		*0.02*	*Ns*	*Ns*	*Ns*	*Ns*	*<0.001*	*0.02*
**Mixed dishes**	**Atassi**	P1	2.9 ± 0.0	1.1 ± 0.1	24.7 ± 0.1	1.1 ± 0.0	126 ± 1	7.0 ± 0.8	0.5 ± 0.0	18.4 ± 1.4	0.5 ± 0.0	123.2 ± 9.7	11.6 ± 1.6	35.1 ± 0
		P2	2.9 ± 0.0	0.2 ± 0.0	23.1 ± 0.0	0.9 ± 0.0	111 ± 0	11.0 ± 0.6	0.2 ± 0.0	9.1 ± 0.5	0.3 ± 0.0	65.4 ± 4.1	9.1 ± 0.3	28.3 ± 0
		P3	3.1 ± 0.1	0.9 ± 0.0	23.3 ± 0.1	1.1 ± 0.0	119 ± 0	6.5 ± 0.4	0.4 ± 0.0	12.8 ± 0.8	0.3 ± 0.1	82.6 ± 6.6	16.6 ± 0.9	39.4 ± 0
		P-value	*0.02*	*<0.001*	*<0.001*	*<0.001*		*0.01*	*<0.001*	*0.01*	*0.02*	*0.01*	*<0.001*	*<0.001*
	**Abla**	P1	4.3 ±0.0	11.3 ±0.0	16.0 ±0.0	1.5 ±0.0	193 ± 0	27.1 ±0.8	1.9 ±0.0	41.0 ±1.1	0.8 ±0.0	204.3 ±6.8	59.1 ±3.8	57.6 ±1
		P2	4.1 ±0.0	8.3 ±0.1	14.2 ±0.1	1.4 ±0.0	158 ± 0	25.5 ±0.3	5.1 ±0.2	38.8 ±0.4	0.7 ±0.0	193.9 ±0.7	41.9 ±4.1	42.3 ±2
		P3	2.5 ±0.0	11.2 ±0.1	10.5 ±0.1	0.9 ±0.0	163 ± 0	22.5 ±0.6	1.9 ±0.0	38.7 ±1.2	0.6 ±0.0	101.8 ±4.9	20.0 ±3.2	50.9 ±3
		P-value	*<0.001*	*<0.001*	*<0.001*	*<0.001*		*0.01*	*<0.001*	*Ns*	*0.01*	*<0.001*	*<0.001*	*0.02*
	**Djongoli**	P1	3.2 ± 0.1	7.8 ± 0.2	16.5 ± 0.3	1.4 ± 0.0	160 ± 1	7.3 ± 0.5	1.3 ± 0.3	30.3 ± 0.9	0.7 ± 0.0	145.4 ± 3.4	14.9 ± 2.6	33.1 ± 1
		P2	5.8 ± 0.0	9.7 ± 0.2	21.8 ± 0.2	1.1 ± 0.0	208 ± 1	12.1 ± 0.4	1.8 ± 0.1	45.0 ± 1.9	1.0 ± 0.0	256.6 ± 12.1	96.5 ± 21.8	50.4 ± 2
		P3	3.7 ±0.0	4.0 ±0.0	17.7 ±0.0	1.3 ±0.0	132 ± 0	10.2 ±0.2	1.6 ±0.1	36.3 ±0.7	0.7 ±0.0	178.1 ±4.5	32.5 ±4.1	49.4 ±3
		P-value	*<0.001*	*<0.001*	*<0.001*	*<0.001*		*0.002*	*Ns*	*0.003*	*0.007*	*0.001*	*<0.001*	*0.005*

Values are the mean ± standard deviation (SD) (n=2 or 3 in case of folate) and p-values are provided when significant (p <0.05).

Table S6. Nutritional densities of the nine cowpea-based dishes (per 100 kcal).

	Dish	Protein (g)	Fat (g)	Available carbohydrate (g)	Dietary fibre (g)	Ash (g)	Ca (mg)	Mg (mg)	K (mg)	Fe (mg)	Zn (mg)	Folate (µg)	Thiamine (µg)
**Stews**	Abobo	6.4 ±0.3	0.5 ±0.1	14.4 ±0.8	6.0 ±0.8	1.6 ±0.6	31.3 ±7.1	40.1 ±5.5	276 ±27	1.4 ±0.4	1.0 ±0.1	71.1 ±15	44.3 ±13
	Vêyi	6.4 ±0.2	0.5 ±0.1	14.3 ±0.6	6.2 ±0.5	1.6 ±0.1	23.8 ±2.5	40.1 ±5.2	280 ±46	1.3 ±0.2	1.0 ±0.1	69.1 ±4	77.8 ±37
	Adowè	6.6 ±0.3	0.5 ±0.2	14.6 ±0.6	5.4 ±0.3	1.6 ±0.3	27.2 ±5.8	39.5 ±2.4	277 ±32	1.1 ±0.1	1.0 ±0.1	69.2 ±20	27.9 ±20
**Doughnuts**	Ata	2.7 ±0.6	6.6 ±1.1	6.7 ±1.7	1.7 ± 0.3	0.7 ±0.2	9.7 ±1.0	17.8 ±3.4	120 ±27	1.5 ± 0.3	0.4 ± 0.1	25.9 ±10	47.6 ±12
	Ata-doco	3.3 ±1.0	5.6 ±1.6	8.1 ±2.2	2.3 ±0.5	0.8 ±0.2	12.1 ±4.9	22.3 ±6.2	158 ±52	1.6 ±0.8	0.5 ±0.1	22.6 ±4	48.1 ±4
	Ataclè	2.2 ±0.2	7.4 ±0.4	5.4 ±0.7	1.6 ±0.1	0.5 ±0.1	7.5 ±1.9	15.4 ±2.2	112 ±12	1.9 ±1.7	0.4 ±0.1	19.0 ±6	39.3 ±7
**Mixed dishes**	Atassi	2.5 ±0.2	0.6 ±0.3	20.0 ±0.7	2.3 ±0.1	0.9 ±0.1	7.0 ±2.3	11.2 ±2.9	75 ±19	0.3 ±0.1	0.3 ±0.1	10.3 ±2.5	28.8 ±3
	Abla	2.1 ±0.5	6.0 ±0.7	7.9 ±1.2	3.0 ±0.3	0.8 ±0.2	14.7 ±1.2	23.2 ±1.6	97 ±28	1.8 ±1.1	0.4 ±0.1	22.0 ±11	29.3 ±2
	Djongoli	2.5 ±0.4	4.2 ±0.9	11.4 ±1.5	3.2 ±0.6	0.8 ±0.2	6.0 ±1.4	22.7 ±3.9	117 ±21	0.9 ±0.2	0.5 ±0.1	26.8 ±17	27.5 ±8

Values are the mean ± SD.

Table S7. Daily contributions to the RNIs of the nine cowpea-based dishes among cowpea consumers* during the week before the FFQ survey (%).

Area	Group	Dish	Protein (%)	Dietary fibre (%)	Ca (%)	Fe (%)	Mg (%)	Zn (%)	K (%)	Folate (%)	Thiamine (%)
Rural	***Stews***	*Abobo*	10.9 [9.9; 11.9]	18.1 [16.4; 19.7]	2.9 [2.6; 3.1]	3.0 [2.7; 3.3]	15.4 [13.9; 16.8]	7.1 [6.4; 7.7]	7.2 [6.6; 7.9]	13.3 [12.1; 14.6]	3.5 [3.2; 3.8]
		Vêyi	1.2 [0.8; 1.6]	2.1 [1.4; 2.8]	0.2 [0.2; 0.3]	1.3 [1.1; 1.6]	1.7 [1.2; 2.3]	0.8 [0.6; 1.1]	0.8 [0.6; 1.1]	1.8 [1.2; 2.4]	0.9 [0.6; 1.2]
		Adowè	0.1 [0.1; 0.2]	0.2 [0.1; 0.3]	0.03 [0.01; 0.05]	0.03 [0.01; 0.05]	0.2 [0.1; 0.3]	0.1 [0.04; 0.1]	0.1 [0.04; 0.1]	0.2 [0.1; 0.3]	0.05 [0.02; 0.08]
	Dough nuts	*Ata*	1.0 [0.8; 1.3]	1.1 [0.9; 1.4]	0.2 [0.1; 0.3]	0.7 [0.5; 0.9]	1.5 [1.1; 1.9]	0.7 [0.5; 0.9]	0.7 [0.5; 0.9]	1.4 [1.0; 1.8]	0.8 [0.6; 0.9]
		Ata-doco	1.7 [1.4; 2.0]	2.1 [1.7; 2.5]	0.3 [0.3; 0.4]	1.0 [0.9; 1.2]	2.6 [2.1; 3.1]	1.2 [1.0; 1.5]	1.2 [1.0; 1.5]	1.6 [1.3; 1.9]	1.2 [0.9; 1.4]
		Ataclè	0.3 [0.1; 0.4]	0.3 [0.1; 0.5]	0.05 [0.02; 0;07]	0.3 [0.1; 0.4]	0.4 [0.2; 0.6]	0.2 [0.1; 0.3]	0.2 [0.1; 0.3]	0.3 [0.1; 0.5]	0.2 [0.1; 0.3]
	Mixed dishes	*Atassi*	5.9 [5.4; 6.4]	9.6 [8.8; 10.5]	0.9 [0.8; 1.0]	0.9 [0.8; 1.0]	5.9 [5.4; 6.4]	3.1 [2.9; 3.4]	2.7 [2.5; 3.0]	3.3 [3.0; 3.6]	3.3 [3.0; 3.5]
		Abla	0.5 [0.3; 0.6]	1.2 [0.8; 1.6]	0.2 [0.1; 0.2]	0.5 [0.3; 0.7]	1.2 [0.8; 1.7]	0.4 [0.3; 0.6]	0.3 [0.2; 0.4]	0.7 [0.5; 0.9]	0.4 [0.2; 0.5]
		Dongola	2.9 [2.3; 3.4]	6.5 [5.3; 7.7]	0.4 [0.3; 0.4]	0.3 [0.2; 0.4]	5.8 [4.7; 6.9]	2.5 [2.0; 2.9]	2.0 [1.7; 2.4]	4.2 [3.4; 4.9]	1.1 [0.9; 1.3]
Urban	***Stews***	*Abobo*	9.9 [9.0; 10.9]	16.5 [14.9; 18.1]	2.6 [2.4; 2.9]	2.8 [2.5; 3.0]	14.0 [12.7; 15.4]	6.5 [5.8; 7.1]	6.6 [6.0; 7.2]	12.2 [11.0; 13.2]	3.2 [2.9; 3.5]
		Vêyi	2.7 [2.1; 3.3]	4.7 [3.7; 5.7]	0.5 [0.4; 0.7]	0.7 [0.5; 0.8]	3.8 [3.0; 4.7]	1.8 [1.4; 2.2]	1.8 [1.4; 2.2]	4.0 [3.1; 4.8]	1.5 [1.2; 1.9]
		Adowè	0.6 [0.5; 0.8]	0.9 [0.7; 1.1]	0.5 [0.4; 0.7]	0.1 [0.1; 0.2]	0.8 [0.6; 1.1]	0.4 [0.3; 0.5]	0.4 [0.3; 0.5]	0.9 [0.7; 1.1]	0.1 [0.1; 0.2]
	Dough nuts	*Ata*	5.1 [1.9; 3.9]	5.7 [4.8; 6.5]	3.5 [3.0; 4.1]	3.6 [3.1; 4.1]	7.6 [6.5; 8.7]	3.6 [3.1; 4.1]	3.5 [3.0; 4.0]	7.0 [6.0; 8.0]	4.2 [3.6; 4.8]
		Ata doco	2.9 [1.9; 3.9]	3.6 [2.4; 4.8]	0.6 [0.4; 0.8]	1.7 [1.1; 2.3]	4.5 [3.0; 6.0]	2.2 [1.4; 2.9]	2.1 [1.4; 2.9]	2.8 [1.8; 3.7]	2.1 [1.4; 2.7]
		Ataclè	0.9 [0.3; 1.4]	1.1 [0.4; 1.8]	0.2 [0.1; 0.2]	0.9 [0.3; 1.5]	1.3 [0.5; 2.2]	0.6 [0.2; 1.0]	0.7 [0.2; 1.1]	1.0 [0.4; 1.6]	0.7 [0.3; 1.7]
	Mixed dishes	*Atassi*	5.0 [4.5; 5.4]	8.1 [7.4; 8.9]	0.7 [0.7; 0.8]	0.8 [0.7; 0.8]	5.0 [4.6; 5.5]	2.7 [2.4; 2.9]	2.3 [2.1; 2.5]	2.7 [2.5; 3.0]	2.7 [2.4; 2.9]
		Abla	0.3 [0.1; 8.0]	0.7 [0.2; 1.2]	0.4 [0.1; 0.7]	0.3 [0.1; 0.5]	0.7 [0.2; 1.2]	0.3 [0.1; 0.4]	0.2 [0.1; 0.3]	0.4 [0.1; 0.7]	0.05 [0.01; 0.1]
		Djongoli	0.9 [0.7; 1.2]	2.1 [1.6; 2.7]	0.1 [0.1; 0.2]	0.4 [0.3; 0.6]	1.9 [1.4; 2.5]	0.8 [0.6; 1.0]	0.7 [0.5; 0.7]	1.4 [1.0; 1.8]	0.5 [0.3; 0.6]

Values are the mean [95% confidence intervals]. *All respondents with the exclusion of non-consumers of cowpea seeds.

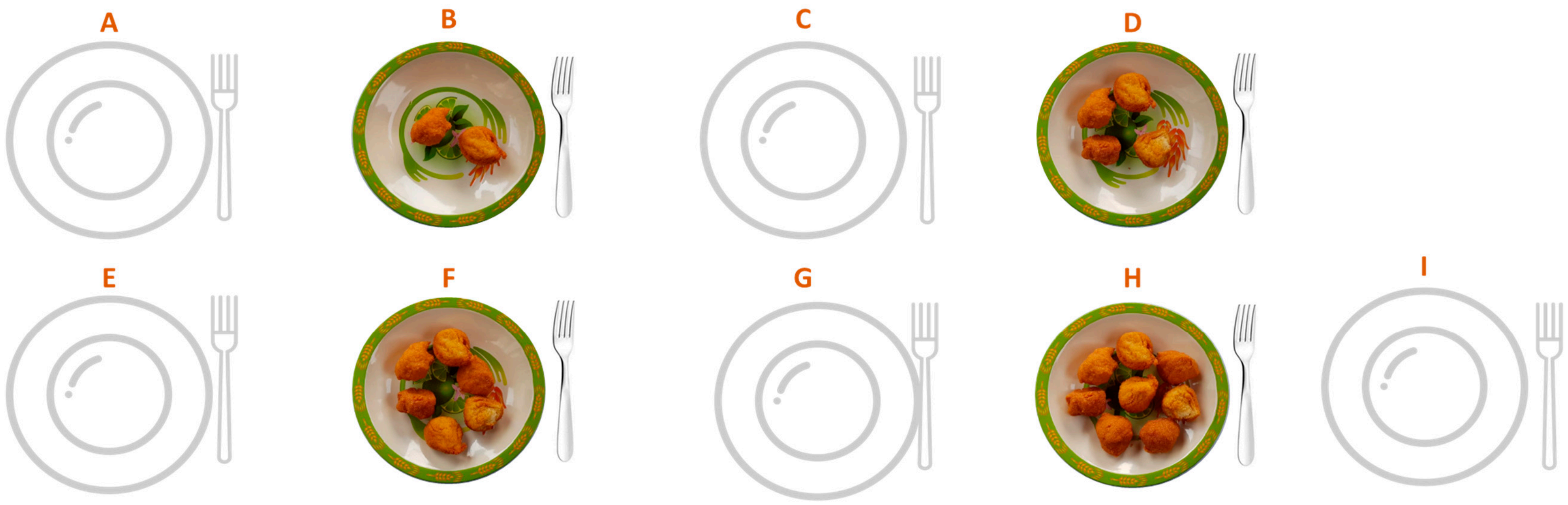

Figure S1. Example of portion sizes for the cowpea-based doughnuts Ata presented in the food atlas.

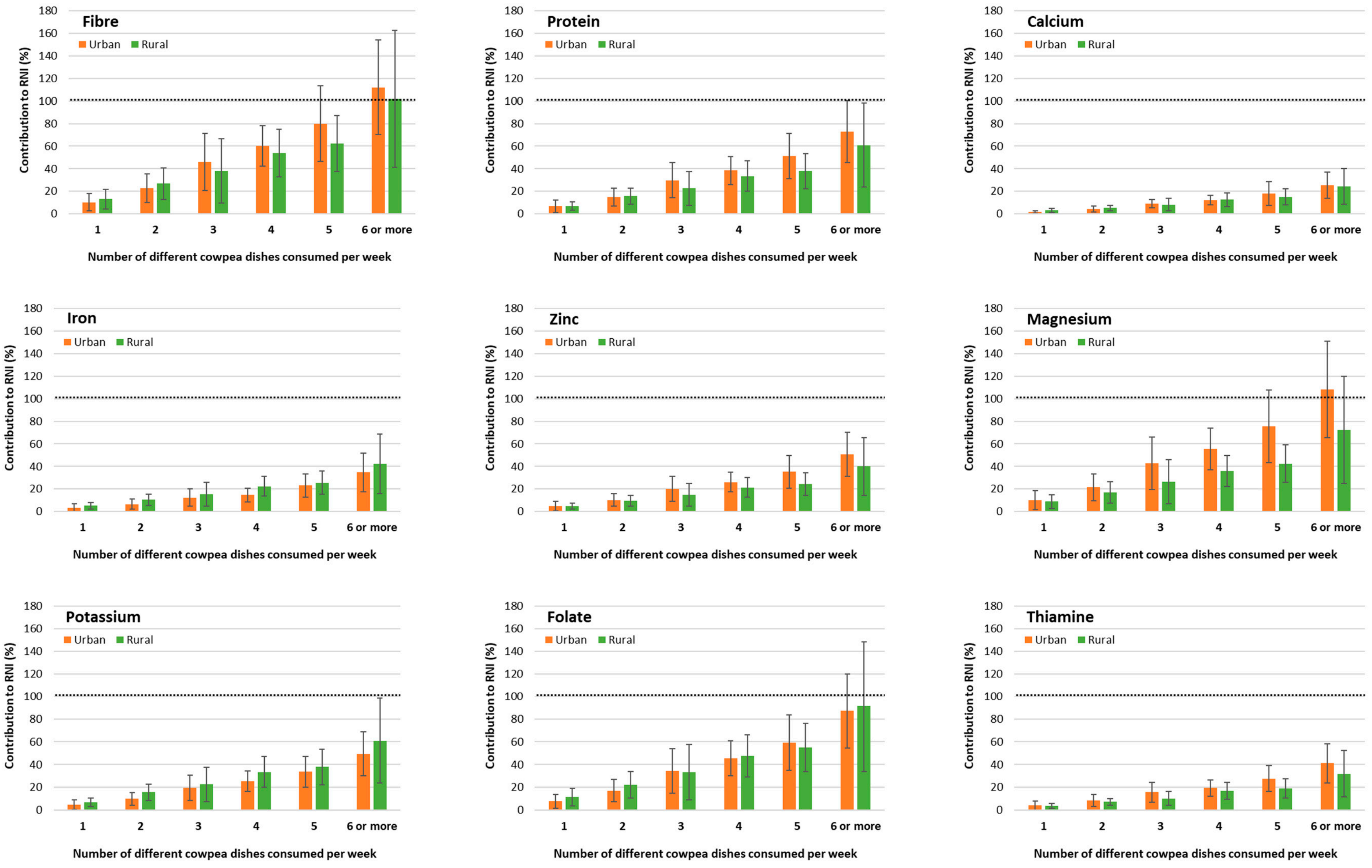

Figure S2. Estimation of the contribution of cowpea-based dishes to RNI, depending on the number of different dishes consumed in the week preceding the FFQ survey

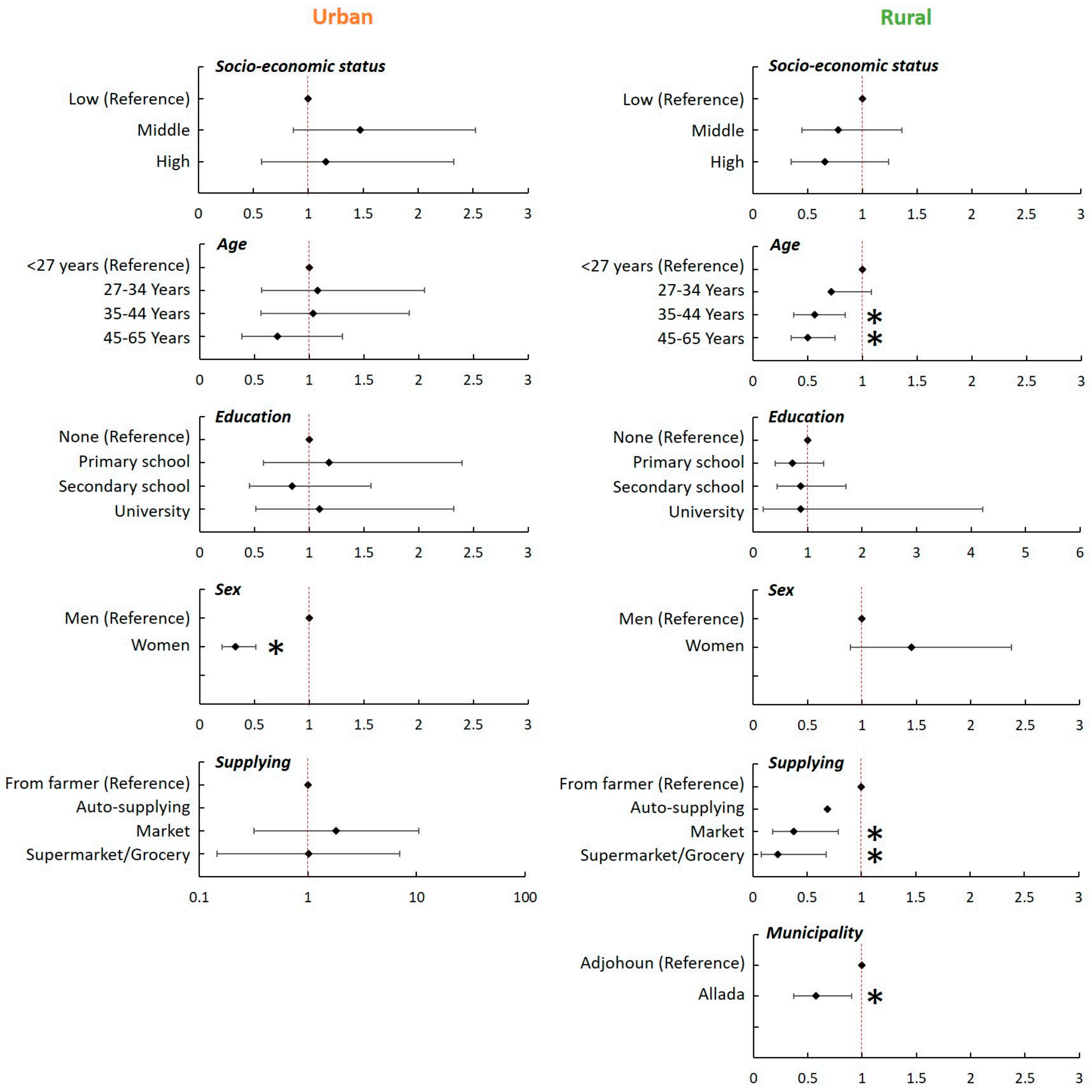

Figure S3. Influence of socio-economic and demographic factors on the chance to meet the Eat-lancet Commission recommendation for daily legume intake (set at 50 g): odd ratios. For consumers with daily cowpea intake lower than the 50 g threshold, we attributed the modality "0", while for consumers who consumed more than 50 g, we assigned the modality "1". Dashed vertical red lines represent the reference for each factor. *: p value < 0.05.
